# Ten quick tips for causal analysis of biomedical omics data

**DOI:** 10.1371/journal.pcbi.1014668

**Published:** 2026-08-20

**Authors:** Gleb Svinin, Rebecca Ting Jiin Loo, Nikhilesh Vasantha Kumar, Varsha Venkatesha Murthy, Dilara Uzuner Odongo, Ramón Díaz-Uriarte, Ana Conesa, Gianluca Bontempi, Susana Vinga, Ilaria Granata, Daniel Domingo-Fernández, Paola Lecca, Marieke L. Kuijjer, Jesse H. Krijthe, Ina Koch, Laurence Calzone, Simona Ester Rombo, Fátima Sánchez-Cabo, Enrico Glaab

**Affiliations:** 1 Biomedical Data Science Group, Luxembourg Centre for Systems Biomedicine (LCSB), University of Luxembourg, Esch-sur-Alzette, Luxembourg; 2 Instituto de Investigaciones Biomédicas Sols-Morreale (IIBM), UAM-CSIC, Madrid, Spain; 3 Department of Biochemistry, Universidad Autónoma de Madrid, Madrid, Spain; 4 Institute for Integrative Systems Biology, Spanish National Research Council, Valencia, Spain; 5 Machine Learning Group, Université Libre de Bruxelles, Bruxelles, Belgique and WEL Research Institute, Wavre, Belgique; 6 INESC-ID, Instituto Superior Técnico, Universidade de Lisboa, Lisbon, Portugal; 7 Institute for High Performance Computing and Networking, National Research Council, Naples, Italy; 8 Fraunhofer Institute for Algorithms and Scientific Computing, Sankt Augustin, Germany; 9 Faculty of Engineering, Free University of Bozen-Bolzano, Bolzano, Italy; 10 Faculty of Medicine, University of Helsinki, Helsinki, Finland; Norwegian Centre for Molecular Biosciences and Medicine (NCMBM), University of Oslo, Oslo, Norway; 11 Pattern Recognition & Bioinformatics, Delft University of Technology, Delft, The Netherlands; 12 Institute of Computer Science, Goethe University Frankfurt, Frankfurt, Germany; 13 Institut Curie, Inserm U1331, PSL, Mines ParisTech, Paris, France; 14 Department of Mathematics and Computer Science, University of Palermo, Palermo, Italy; 15 Centro Nacional de Investigaciones Cardiovasculares (CNIC), Madrid, Spain; Max Delbruck Centre for Molecular Medicine: Max-Delbruck-Centrum fur Molekulare Medizin in der Helmholtz-Gemeinschaft, GERMANY

## Introduction

Omics data generation has become routine in biomedical research. Transcriptomics, proteomics, and metabolomics now produce datasets of considerable scale and resolution. Integrating these diverse modalities requires carefully addressing common analytical pitfalls [1]. A standard analysis involves statistical comparisons between conditions (e.g., disease versus control), providing ranked lists of differentially abundant molecules. While informative for biomarker discovery, these lists describe associations rather than mechanisms: they indicate what changes but not what causes those changes or how interventions might alter the system’s behavior [2–4].

Causal inference methods aim to go beyond correlations by modeling directed regulatory influences, identifying candidate upstream drivers of disease phenotypes, and predicting the effects of molecular interventions. They range from counterfactual frameworks and graphical causal models to logic-based representations of regulatory circuits [5,6]. These frameworks address the following distinct tasks: causal discovery (learning structure from data), identification (expressing a causal quantity in terms of the observed data distribution under stated assumptions), estimation (fitting the relevant statistical relationships), and inference (quantifying uncertainty of estimated effects), so that the relevant references and tools differ by task. While some omics studies use controlled lab experiments, many rely on observational data from clinical cohorts or existing biobanks. In these observational settings, researchers do not assign treatments or alter the biology themselves, meaning causal reasoning is necessary to clarify what the data can actually answer and to help manage the limits of the study design. The degree of experimental control, ranging from observational cohorts to designed perturbation screens, further determines how the tips below should be read. Applying these methods to biomedical data requires careful attention to study design, data quality, prior biological knowledge, and the assumptions inherent in each framework, as well as rigorous initial data analysis [7].

**Fig 1:**
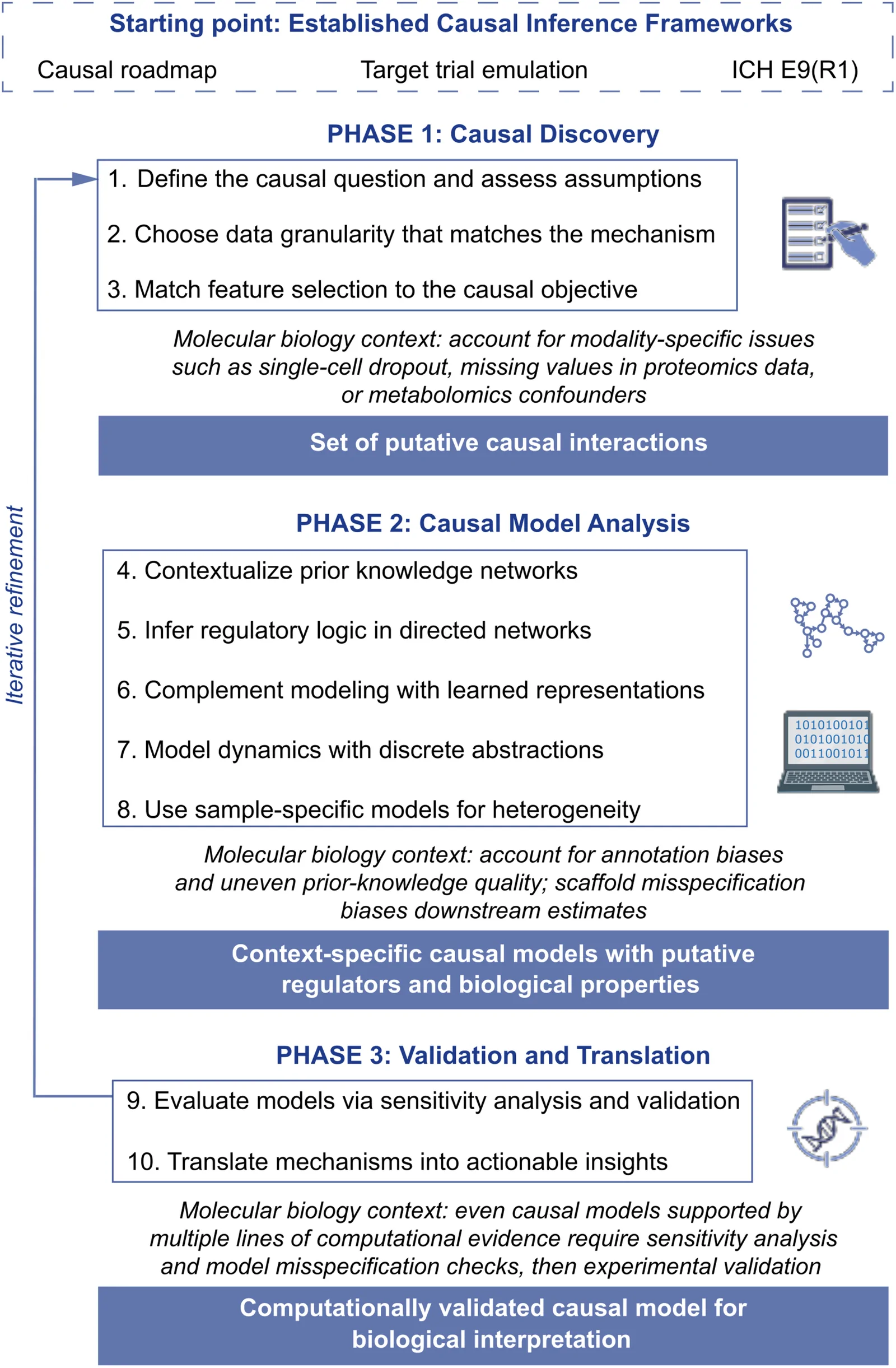
Overview of the ten tips organized by analysis phase. The tips are grouped into three phases: Phase 1 (Causal Discovery, Tips 1–3), Phase 2 (Causal Model Analysis, Tips 4–8), and Phase 3 (Validation and Translation, Tips 9–10). Solid arrows indicate the primary workflow; the arrow from validation back to Phase 1 reflects the iterative nature of causal analysis. The workflow takes the causal roadmap of statistical and causal inference [8] and target trial emulation [9], together with the ICH E9(R1) estimand addendum, as its starting point, and then marks the steps that require field-specific adaptation in molecular biomedicine, such as adapting the estimand to the molecular entities under study, choosing data granularity, contextualizing prior-knowledge networks, and validating predictions experimentally.

The ten quick tips presented here were compiled from the insights shared during an international lecture series on ‘Causal Analysis of Biomedical Data’ held at the University of Luxembourg in 2024/2025, given by researchers with complementary expertise spanning causal inference, network biology, Boolean modeling, and clinical translation. While no single set of suggestions can address every scenario, this guide provides a practical roadmap for moving from molecular associations to causal, mechanistic understanding (Fig 1). The tips are organized into three phases: causal discovery (Tips 1–3), causal model analysis (Tips 4–8), and validation and translation (Tips 9–10).

### Tip 1: Define the causal question and assess assumptions before collecting data

Before collecting data, researchers should formulate the specific causal question the study is intended to address. Whether the aim is to estimate the effect of a molecular intervention, identify an upstream cause of a disease phenotype, or address a counterfactual question (see [6,10] for the distinction between interventions and counterfactuals), the question helps to determine which data and methods are appropriate. Prediction and causal inference should not be conflated: a gene expression signature that accurately predicts tumor recurrence may reflect downstream effects rather than causal drivers [4], and a model predicting cardiovascular risk does not identify the main risk factor for a specific individual [11]. Formally, causal questions concern the consequences of interventions rather than conditional associations [6,12].

To support the intended causal inference, researchers should make their assumptions about the causal structure explicit, including potential confounders. These assumptions can be represented in a directed acyclic graph (DAG), which helps assess whether the available data can support the intended causal claim and reveals potential sources of bias [2,12,13]. If relationships between variables are not carefully considered, paradoxical conclusions may arise, for example, when a treatment appears to increase risk because treated patients already had worse prognosis [5,14]. It is important to separate two kinds of assumptions: identification assumptions, which determine whether a causal estimand can be written as a function of the observed data distribution given a DAG, and statistical or estimation assumptions, which concern the models used to fit the resulting relationships, including assumptions about nuisance functions [8,15]. Three identification assumptions warrant particular attention: exchangeability (absence of unmeasured confounding), positivity (non-zero probability of each intervention level in all subgroups), and consistency (well-defined interventions). These three conditions are sufficient for estimating an average treatment effect under a point intervention but are not universal: other designs require additional or alternative assumptions. For example, Mendelian randomization in population genetics relies on instrumental-variable assumptions [16], whereas g-methods for longitudinal and survival settings with time-varying exposures require sequential exchangeability [12]. Identification assumptions differ in the extent to which they can be examined empirically. Exchangeability and consistency cannot be verified from the observed data, whereas positivity, that is, sufficient overlap between the intervention groups, can be diagnosed in practice, for example, by inspecting propensity score distributions [17]. When the aim is to estimate an effect rather than to recover structure, doubly robust and semiparametric efficient estimators, including augmented inverse-probability weighting, one-step estimators, and targeted maximum likelihood estimation, reduce sensitivity to misspecification of any single nuisance model [15,18,19]. In turn, defining the estimand before analysis helps guide subsequent analytical decisions [20]. This step is formalized by established frameworks, in particular target trial emulation [9] and the causal roadmap for generating real-world evidence [8], which builds on the statistical causal modeling framework [15] and has been taken up by health technology assessment and regulatory bodies, including the ICH E9(R1) estimand addendum [21]. These frameworks provide a structured starting point for molecular studies, with the field-specific challenges addressed in the following tips separately.

### Tip 2: Choose data granularity that matches the mechanism

Causal signals can be obscured by inappropriate aggregation. Coarse gene-level quantification may overlook isoform-switching events with distinct functional consequences [22]. Inference of evolutionary accumulation models from bulk data may reconstruct composite genotypes that do not correspond to any real genotype, because mutations from different cell populations are merged [23]. More generally, bulk averaging can mask signals present only in minority cell populations.

Whether aggregation is a problem depends on the target estimand defined in Tip 1. If the question concerns a population-average effect, heterogeneous effects that average out across cells or subgroups are the intended quantity rather than an error; granularity matters when the question instead concerns cell-type-specific or subgroup-specific mechanisms that aggregation would obscure. Data granularity should therefore match the hypothesized causal mechanism. Long-read sequencing can reveal disease-associated isoform switches missed by standard short-read RNA-seq [24]. If the causal hypothesis involves cell-type-specific regulation, single-cell or spatial transcriptomics may be required; some methods, such as TENET [25], infer causal relationships directly from single-cell measurements. Others, including the methods discussed in Tip 5, can take pseudobulk-derived fold changes as input, which improves robustness in settings where single-cell resolution is not needed. If protein function is central, measuring post-translational modifications may be more informative than aggregate gene expression. The greater cost and complexity of higher-resolution assays must be balanced against the risk of losing causal signals through aggregation. Practical challenges and plausibility of assumptions also differ across omics modalities, even when the underlying causal framework is unchanged: single-cell transcriptomics is affected by dropout and sparsity, proteomics often presents values missing not at random and typically achieves lower proteome coverage, and metabolomics is sensitive to environmental and dietary confounders. These differences mainly affect preprocessing choices and which confounders must be considered, and should be weighed alongside the granularity considerations above.

### Tip 3: Match feature selection to the causal objective

High-dimensional, multiscale biomedical data involve statistical associations that do not reflect causal relationships. For mechanistic understanding and intervention-oriented analysis, feature selection should distinguish causes from effects and indirect associations [26]. A differentially expressed gene at the bottom of a regulatory cascade is less informative for intervention design than an upstream regulator with a smaller effect size.

Causal feature selection methods address this challenge using strategies distinct from standard correlation-based filtering. Information-theoretic approaches use asymmetric descriptors of inter-variable relationships, or exploit auxiliary variables to clarify the relationship among variables of interest. One such method [26] prioritizes putative direct causes of a target variable by filtering out features whose association with the outcome is fully explained by other features. Subsequent extensions [27] handle multivariate outcomes, while further work [28] infers pairwise causal relationships using asymmetric descriptors, moving closer to network reconstruction. Complementary to this, structured sparsity and network-based penalty terms in regularized regression can incorporate prior knowledge of regulatory relationships, highlighting upstream features within signaling pathways [29,30]. These causal feature selection methods rely on assumptions such as faithfulness and valid conditional-independence testing, but these can be difficult to justify in high-dimensional omics data. Faithfulness can fail, for example, when effects along distinct causal paths cancel, making causally connected variables appear statistically independent and causing true edges to be missed. Under model misspecification or violated assumptions, both false positives and false negatives can arise, and explicit error-rate control is still an open problem (a benchmark survey covers available methods and their failure modes [31]). Such outputs are therefore best treated as hypotheses for downstream validation rather than definitive causal claims.

### Tip 4: Contextualize prior knowledge networks

Purely data-driven approaches for learning causal network structure are often underpowered in biomedical settings, where sample sizes are small relative to the number of variables [32]. Prior Knowledge Networks (PKNs) from curated databases provide a biologically informed starting point, and different PKN resources offer complementary strengths: SIGNOR [33] provides directed, signed causal relationships between bioentities, Reactome [34] organizes molecular interactions into pathway maps, and OmniPath [35] integrates over 100 resources into a single compilation. However, these databases contain interactions observed across many conditions, producing dense, non-context-specific networks.

A practical strategy for addressing this issue is to contextualize PKNs using experimental data [36,38]. Nodes not expressed in the tissue or condition of interest are removed along with their associated edges, while recognizing that absence of detection does not always imply biological irrelevance. Tools such as PathMe [36] and NeKo [38] enable integration of mechanistic pathway knowledge from multiple resources, automating the construction of context-specific subnetworks. A complementary strategy is penalized regression, which uses graph-aware penalties derived from PKNs to identify condition-associated subnetworks by encouraging similar regression coefficients across connected genes [29,30]. However, careful attention should be paid to quality, coverage, and potential biases of the underlying databases, as missing or incorrect interactions may propagate into the model. More broadly, when a contextualized network is used as the scaffold for causal or regulatory inference, misspecification of this scaffold relative to the unknown ground truth biases downstream estimates, which is one reason the robustness checks in Tip 9 are needed.

### Tip 5: Infer regulatory logic in directed networks

In correlation networks, edges indicate that two variables are associated but not which one influences the other. Because no direction of effect is encoded, such networks cannot propagate signals from putative causes to downstream effects. Directed interaction networks, in which edges are oriented from source to target, overcome this limitation. As discussed in Tip 4, a variety of directed prior-knowledge networks are available, providing a basis for tracing regulatory effects through the system.

These directed networks can be combined with experimental data to infer regulatory activity and formulate signaling hypotheses. VIPER [39], for example, estimates regulator activity by aggregating expression changes across a regulon, i.e., the set of a regulator’s known target genes. CARNIVAL [40] identifies consistent signaling paths within a signed, directed PKN by integrating perturbation-response footprints. By combining these data-driven approaches with curated databases for transcription factor–target relationships, such as DoRothEA [41], researchers can maximize the coverage of directed, signed edges in their networks. The resulting models make explicit predictions about which regulators are active or inactive under a given condition, directly informing causal hypothesis generation. The reliability of predictions depends on the quality and completeness of the underlying regulons and prior knowledge networks, and footprint-based activity estimates assume that the measured downstream changes reflect the activity of the proposed regulator. Where these assumptions are only partially met, independent benchmarking and experimental validation are important before the inferred activities are interpreted causally.

### Tip 6: Complement causal modeling with learned representations

Biological interaction networks are structured objects whose topology encodes functional organization [42]. While embedding such networks into metric spaces is not a causal inference technique per se, it generates topology-aware representations that support downstream analyses informing causal modeling. At the node level, mapping gene networks onto a metric space can uncover latent structural properties. Evaluations have shown that hyperbolic space is particularly well suited for biological networks, as it preserves distances of the original graph more faithfully than Euclidean alternatives [43]. In these embeddings, standard clustering techniques can identify centers of information spreading, providing a mathematically grounded approach to nominating candidate drivers of network dynamics.

At the whole-graph level, frameworks like Netpro2vec [37] embed each graph within a collection as a fixed-length vector by summarizing topological features through node distance distributions and transition matrices. These graph-level representations enable direct comparison, clustering, and visualization of multiple networks, for instance to contrast disease and control networks or to identify network subtypes across a patient cohort. Combining node- and graph-level embeddings helps distill complex topological information into features that can guide and refine mechanistic and causal models. When embedding-derived features are used as covariates in downstream causal analyses, for example, in the exposure-assignment model or the outcome model, errors in these representations can propagate into effect estimates. The doubly robust estimators described in Tips 1 and 9 can reduce sensitivity to misspecification of one of these nuisance models, although they do not improve the embedding itself. A related and rapidly developing direction is causal representation learning, which aims to recover latent causal variables and their relations from high-dimensional measurements rather than from predefined features [44]. Steps in this direction for omics include methods that identify regulatory edges which change between conditions such as disease and control states [45], though a general implementation of causal representation learning for high-dimensional omics remains an active area of development. These methods address a distinct but related challenge: rather than representing known topology in metric spaces, they aim to recover latent causal structure directly from high-dimensional data, with their assumptions and identifiability conditions still being actively clarified.

### Tip 7: Model system dynamics using discrete abstractions

Frameworks for modeling biological dynamics can be broadly divided into kinetic and non-kinetic approaches. Kinetic models provide quantitative predictions but require extensive parametrization, with reaction rate constants often unavailable at the quality needed for reliable inference in larger systems [46]. Non-kinetic approaches offer an alternative by capturing qualitative system behavior without detailed kinetic parameters.

One non-kinetic formalism is Boolean modeling, in which each biological entity is represented by a binary state (active or inactive) and its state at the next step is determined by logical rules encoding regulatory dependencies. These models can recapitulate stable states (attractors) of signaling pathways and predict qualitative effects of perturbations. The field includes many variants, including frameworks such as MaBoSS [47,48] that use stochastic continuous-time simulations to produce probabilistic predictions of attractor state distributions, without requiring explicit kinetic rate parameters. The CoLoMoTo consortium provides a unified environment for Boolean network analysis [49]. Another discrete formalism is Petri nets, which represent networks as bipartite graphs to distinguish between passive components (places, e.g., molecules) and active components (transitions, e.g., reactions), and describe system dynamics using movable tokens [50]. For Petri net modeling and analysis, the software MonaLisa is available [51]. Overall, these discrete approaches are particularly useful for studying knockout or overexpression effects, assessing state reachability, and identifying steady states corresponding to known disease phenotypes.

### Tip 8: Account for biological heterogeneity via sample-specific models

A single consensus network inferred from a disease cohort averages over patients and can mask differences in underlying mechanisms. However, causal drivers may differ across individuals due to genetic background, environmental exposures, disease stage, and comorbidities. This matters in therapeutic settings, where a drug target may be relevant only for a subset of patients [52].

Methods for constructing sample-specific networks address this limitation. LIONESS [53,54] derives a network for each sample by computing the change in edge weights between the aggregate network inferred from all samples and the network obtained when that sample is removed. By contrast, PatientProfiler [55] generates sample-specific networks by integrating prior-knowledge interactions with individual sample data. The resulting single-sample networks support topology-aware downstream analyses: patients can be grouped based on similarity between their inferred networks and the resulting groups can then be tested for differences in clinical outcomes such as survival [55]. Collections of individual weighted networks can also be used to identify condition-specific network patterns [56]. More recent work has applied large-scale causal discovery using interventional data to shed light on gene network structure [57]. When full patient-specific modeling is not feasible, stratifying the cohort by known clinical variables before network construction can still help capture mechanistic heterogeneity.

### Tip 9: Evaluate causal models through sensitivity analysis and biological validation

Causal analysis depends on assumptions and prior knowledge. Its conclusions should therefore be subjected to sensitivity analyses and checked for robustness under reasonable alternatives, such as different confounder sets, hyperparameters, or prior-knowledge inputs, to assess whether the findings depend strongly on model configuration and assumptions. When possible, diagnostic checks should be used to assess whether the model was applied appropriately; such checks can, for example, reveal systematic biases in estimated treatment effects. In computational settings, credibility is further strengthened when similar conclusions can be reproduced in an independent dataset, ideally from a different modality. Robustness checks should also probe model misspecification directly, for example, by comparing estimators that rely on different working models, or, where suitable, by using doubly robust estimators whose validity holds if at least one of the nuisance models is correctly specified [2,12,15].

Beyond computational checks, causal hypotheses should be assessed against existing biological knowledge and, ultimately, tested experimentally. For gene regulatory networks, CRISPR-based perturbation screens can test whether predicted regulators induce expected changes in their downstream targets. Discrepancies between predicted and observed effects highlight areas where the model, its assumptions, or its inputs need revision, making the process explicitly iterative (Fig 1). Databases such as the Connectivity Map [58] provide perturbation response profiles for systematic *in silico* validation before generating new predictions. Taken together, sensitivity analysis, biological assessment, and perturbation experiments provide stronger support for causal claims.

### Tip 10: Translate causal mechanisms into actionable insights

The goal of causal analysis in biomedicine is to produce findings that inform clinical decisions, guide therapeutic development, or advance biological understanding. There are encouraging examples of how the methodological toolkit discussed in this article has been applied in practice. For instance, an approach combining the ideas of tips 4 and 5 was used to study FLT3-ITD-positive acute myeloid leukemia, where subtype-specific roles of the kinase WEE1 were identified and subsequent pharmacological inhibition restored therapy sensitivity [59]. In another application, a precision oncology framework successfully prioritized compounds by their ability to invert tumor-checkpoint activity in gastroenteropancreatic neuroendocrine tumors [60].

Looking ahead, *in silico* clinical trials [61] offer a further perspective on translation, though computational frameworks remain constrained by the knowledge and data used to build them. Causal models can provide an advantage for clinical adoption because their structure encodes mechanistic hypotheses that can be communicated as interpretable explanations. However, researchers should present results as prioritized intervention points with predicted mechanistic effects and acknowledge the model’s assumptions and limitations. The above examples illustrate both the translational potential of causal analyses and the need to remain realistic about what such methods can currently deliver.

## Conclusion

Moving from correlation to causation in molecular biology is not only a statistical exercise; it represents a fundamental shift in how biological understanding and therapeutic development are approached. Our ten quick tips provide a workflow for extracting mechanistic insights from high-dimensional molecular data, emphasizing that successful causal analysis requires attention at every stage: formulating causal questions, selecting data at an appropriate granularity, contextualizing prior biological knowledge, accounting for network structure and dynamics, and translating inferred mechanisms into testable hypotheses.

No single approach is universally optimal; the choice of strategy must be guided by the causal question, data quality, and intended application. The iterative nature of causal analysis, where validation informs model refinement and new experiments, should be viewed as a strength rather than a limitation. These quick tips provide a starting point for researchers entering this field and a reference for those already engaged in causal analysis of biomedical data. The applicability of individual tips also depends on the study design: in purely observational cohorts causal reasoning mainly clarifies what can be inferred, whereas designed perturbation experiments can support stronger causal claims.
